# Stress Tolerance Variations in *Saccharomyces cerevisiae* Strains from Diverse Ecological Sources and Geographical Locations

**DOI:** 10.1371/journal.pone.0133889

**Published:** 2015-08-05

**Authors:** Yan-Lin Zheng, Shi-An Wang

**Affiliations:** 1 College of Mathematics and Systems Science, Shandong University of Science and Technology, Qingdao, 266590, China; 2 Key Laboratory of Biofuels, Shandong Provincial Key Laboratory of Energy Genetics, Qingdao Institute of BioEnergy and Bioprocess Technology, Chinese Academy of Sciences, Qingdao, 266101, China; University of Strasbourg, FRANCE

## Abstract

The budding yeast *Saccharomyces cerevisiae* is a platform organism for bioethanol production from various feedstocks and robust strains are desirable for efficient fermentation because yeast cells inevitably encounter stressors during the process. Recently, diverse *S*. *cerevisiae* lineages were identified, which provided novel resources for understanding stress tolerance variations and related shaping factors in the yeast. This study characterized the tolerance of diverse *S*. *cerevisiae* strains to the stressors of high ethanol concentrations, temperature shocks, and osmotic stress. The results showed that the isolates from human-associated environments overall presented a higher level of stress tolerance compared with those from forests spared anthropogenic influences. Statistical analyses indicated that the variations of stress tolerance were significantly correlated with both ecological sources and geographical locations of the strains. This study provides guidelines for selection of robust *S*. *cerevisiae* strains for bioethanol production from nature.

## Introduction

The budding yeast *Saccharomyces cerevisiae* is a vitally important microorganism for human being, which has been instrumental to baking, brewing, distilling, and wine making since ancient times [1]. More recently, *S*. *cerevisiae* has been used as a platform organism for production of bioethanol from various biomass feedstocks, such as corn starch, sugarcane, and lignocellulose [2,3]. Regardless of what kind of biomass feedstock is used, stressors inhibiting yeast growth and fermentation are generated during fermentation processes. High ethanol concentrations, temperature shocks, and osmotic stress are among the most common stressors during fermentations [4,5]. Strains of *S*. *cerevisiae* resistant to such stressors are desirable for bioethanol production [6].

The yeast *S*. *cerevisiae* is among the most thoroughly studied eukaryotic model organisms at the cellular, molecular, and genetic levels. However, the ecology of *S*. *cerevisiae* has not attracted enough attention until recently. Natural isolates of *S*. *cerevisiae* inhabiting tree exudates in forests were discovered fifteen years ago [7,8]. Subsequent studies proposed the existing of both domesticated populations and wild populations [9]. In recent years, it has been recognized that *S*. *cerevisiae* is a ubiquitous species in nature and it occupies numerous habitats from human-associated environments as well as habitats remote from human activity [10,11]. Five world lineages and eight Chinese lineages have been identified as well as many genetic mosaics strains through investigation of diverse isolates from industrial environments, hospitals, breweries, tree barks, leaves, soil, etc. The identification of clear lineages and mosaics strains offered new insights into genetic structure of *S*. *cerevisiae* [11–13]. Meanwhile, phenotypic variations of these *S*. *cerevisiae* lineages attracted increasing attention [12,14–16]. Wild *S*. *cerevisiae* strains collected from oak exudates present apparent differences from vineyard or sake isolates in phenotypic similarity, growth rate, and stress tolerance to a variety of stressors, such as copper sulfate and freeze [16,17]. The finding of eight Chinese *S*. *cerevisiae* lineages provided novel materials for investigating phenotypic variations and related driving forces in diverse *S*. *cerevisiae* populations.

This study aims to investigate the variations of stress tolerance in novel *S*. *cerevisiae* lineages and the influence of ecology and geography on the varied phenotypes. Considering that high ethanol concentrations, temperature shocks, and osmotic stress are the most common stressors during ethanol fermentation, we evaluated the tolerance of *S*. *cerevisiae* strains to these stressors. The results showed that both ecological sources and geographical locations presented significant correlation with variations of stress tolerance. Overall, the isolates from human-associated environments represented a higher level of stress tolerance compared with those from forests spared anthropogenic influences, which provides guidelines for selection of robust *S*. *cerevisiae* strains for bioethanol production from nature.

## Materials and Methods

### Strains

The panel of *S*. *cerevisiae* strains used in stress tolerance analysis consisted of 34 Chinese forest isolates and 37 Chinese orchard isolates, as well as 12 industrial strains deposited in China General Microbiological Culture Collection Center (CGMCC) (Table 1). The forest and orchard isolates were collected and identified according to the methods described previously [11]. All necessary permits were obtained for the described field studies. The owners of the orchards and the authorities responsible for the forests agreed with the collection of samples and the sampling plan. The geographic locations of strains isolated in China covered Jilin province (latitude and longitude across the sampling sites, 41°35'~42°25', 127°40'~128°16'), Beijing areas (39°26'~41°03', 115°25'~117°30'), Shandong province (35°36'~36°25', 116°58'~119°12'), Xinjing province (41°68'~42°95', 86°06'~90°25'), Shannxi province (34°17', 108°20'), Yunnan province (22°00'~25°34', 100°52'~102°73'), and Hainan province (19°52'~20°02', 109°57'~110°35').

**Table 1:** Strains of *S*. *cerevisiae* used in stress tolerance analysis.

Lineage	Strain	Location	Source	Date
**Orchard group**				
CHN IV	BJPTE	Shunyi, Beijing	Vineyard grape	2008
	BJ3	Shunyi, Beijing	Vineyard grape	2008
CHN VI	AQPT45	Anqiu, Shandong	Vineyard grape	2008
	AQT2	Anqiu, Shandong	Grapevine	2008
CHN VIII	SH28-1	Shanshan, Xinjiang	Vineyard grape	2006
	BJ2	Shunyi, Beijing	Apple in orchard	2008
	SD4	Qufu, Shandong	Vineyard grape	2008
	QFPT43	Qufu, Shandong	Vineyard grape	2008
Clade 1	BJPG4	Shunyi, Beijing	Apple in orchard	2008
	BJPG14	Shunyi, Beijing	Apple in orchard	2008
	BJL8	Shunyi, Beijing	Pear in orchard	2008
	BJL10	Shunyi, Beijing	Pear in orchard	2008
Clade 2	XL3-1	Korla, Xinjiang	Pear in orchard	2006
	XL4	Korla, Xinjiang	Pear in orchard	2006
	XL11-2	Korla, Xinjiang	Pear in orchard	2006
	XL13-2	Korla, Xinjiang	Pear in orchard	2006
	XL14-1	Korla, Xinjiang	Pear in orchard	2006
	XL15-2	Korla, Xinjiang	Pear in orchard	2006
	P10-1	Korla, Xinjiang	Apple in orchard	2006
	P16	Korla, Xinjiang	Apple in orchard	2006
	P16-2	Korla, Xinjiang	Apple in orchard	2006
Sake	YN2	Jinghong, Yunnan	Orange in orchard	2006
	BJL4	Shunyi, Beijing	Pear in orchard	2008
	BJL7	Shunyi, Beijing	Pear in orchard	2008
Wine/European	XJ5	Shanshan, Xinjiang	Vineyard grape	2006
	WA3-4	Shunyi, Beijing	Vineyard grape	2008
Mosaic	XJ4	Shanshan, Xinjiang	Vineyard grape	2006
	XJ6	Korla, Xinjiang	Pear in orchard	2006
	P7-1	Korla, Xinjiang	Apple in orchard	2006
	XJ2	Korla, Xinjiang	Apple in orchard	2006
	XJ3	Korla, Xinjiang	Apple in orchard	2006
	BL6	Jinghong, Yunnan	Pineapple in plantation	2006
	YN1	Jinghong, Yunnan	Pineapple in plantation	2006
	BL11C	Jinghong, Yunnan	Pineapple in plantation	2006
	BJL3	Shunyi, Beijing	Pear in orchard	2008
	JL3	Changbai Mt, Jilin	Peach	2007
	YC77	Changbai Mt, Jilin	Oak bark	2008
**Wild group**				
CHN I	HN6	Bawangling Mt, Hainan	Rotten wood	2007
	HN1	Diaoluo Mt, Hainan	Rotten wood	2007
CHN II	SX2	Qinling Mt, Shaanxi	Oak bark	2006
	SX4	Qinling Mt, Shaanxi	Oak bark	2006
	SX5	Qinling Mt, Shaanxi	Oak bark	2006
	SX6	Qinling Mt, Shaanxi	Oak bark	2006
	SX7	Qinling Mt, Shaanxi	Oak bark	2006
	SX8	Qinling Mt, Shaanxi	Oak bark	2006
CHN III	HN19	Wuzhi Mt, Hainan	Oak bark	2006
CHN IV	BJ18	Dongling Mt, Beijing	Oak bark	2008
	LS26	Dongling Mt, Beijing	Oak bark	2008
	LS541	Dongling Mt, Beijing	Oak bark	2008
	LS542	Dongling Mt, Beijing	Oak bark	2008
	BJ23	Dongling Mt, Beijing	Oak bark	2008
	BJ24	Dongling Mt, Beijing	Oak bark	2008
	BJ20	Dongling Mt, Beijing	Oak bark	2008
CHN V	HN17	Wuzhi Mt, Hainan	Oak bark	2006
	HN18	Wuzhi Mt, Hainan	Oak bark	2006
	HN12	Hainan, China	Rotten wood	2007
	HN15	Hainan, China	Soil	2007
	HN16	Hainan, China	Soil	2007
CHN VI	AQSZ8	Anqiu, Shandong	Oak bark	2008
	AQSZ30	Anqiu, Shandong	Oak bark	2008
	SD2	Tai’an, Shandong	Oak bark	2008
Sake	TABL2	Tai’an, Shandong	Oak bark	2008
	JL2	Changbai Mt, Jilin	Oak bark	2008
Mosaic	SX9	Qinling Mt, Shaanxi	Wild tomato	2006
	SX11	Qinling Mt, Shaanxi	Oak bark	2006
	YN6	Zixi Mt, Yunnan	Oak bark	2006
	YDX4	Dali, Yunnan	Soil	2008
	YN5	Dali, Yunnan	Soil	2008
	BJ3P	Haidian, Beijing	A peach flower	2006
	TAHT7	Tai’an, Shandong	Oak bark	2008
	SD3	Tai’an, Shandong	Oak bark	2008
**Industrial group**				
Clade 3	AS2.2	England	Beer	Pre-1952
	AS2.3	England	Beer	Pre-1952
	AS2.4	England	Beer	Pre-1952
Sake	AS2.666	Guizhou, China	Distilled spirit	Pre-1960
Wine/European	AS2.24	England	Brewary	Pre-1952
	AS2.521	Unknown	Ratafee	Pre-1960
	AS2.526	Unknown	Distilled spirit	Pre-1960
	AS2.1422	Japan	Distilled spirit	1955
Mosaic	AS2.381	Russia	Paper mill	Pre-1960
	AS2.383	Russia	Paper mill	Pre-1960
	AS2.395	Russia	Paper mill	Pre-1960
	AS2.1427	Japan	Distilled spirit	Pre-1943

Abbreviation: Mt, mountain.

### Sequence analysis

Four housekeeping genes (*ACT1*, *RPB1*, *RPN2*, and TBP) and three stress response genes (*HSP104*, *HXK1* and *TPS1*) were sequenced in this study [18–21]. Three of the four housekeeping genes (*ACT1*, *RPB1* and *RPN2*) were previously used to infer the diversity of *S*. *cerevisiae* [22, 23]. Gene sequences were amplified using the primer pairs as described in S1 Table. The gene amplification conditions were as follows: 4 min initial denaturation at 94°C, 30 cycles each of 30 sec at 94°C, 45 sec at 52°C or 55°C, and 90 sec at 72°C, and a final extension period of 8 min at 72°C. Population genetic analysis was completed using DnaSP software [24]. McDonald and Kreitman test was performed to evaluate the neutral evolution for the genes [25]. Neighbor-joining tree was constructed from the evolutionary distance data calculated from Kimura’s two-parameter model [26].

### Population structure analysis

Population structure was inferred using the program Structure 2.3.2 [27] based on 143 SNPs, for which neutral evolution was not rejected based on McDonald & Kreitman test. Admixture model with correlated allele frequencies was used. The result was visualized using Distruct 1.1 [28].

### Culture conditions

All the strains were initially grown on YPD slants (10 g L^-1^ of yeast extract, 20 g L^-1^ of peptone, 20 g L^-1^ of dextrose and 20 g L^-1^ of agarose, pH 6.0) for 3 days at 30°C. Afterwards, a loop of each of the colonies was inoculated into 3 mL of YPD broth and cultured for 48 h at 30°C with shaking at 200 rpm. In order to invigorate all the strains, 20 μL of each YPD broth culture was transferred into a new YPD broth tube and cultured for an additional 24 h at 30°C with shaking at 200 rpm. The optical density at 600 nm (OD_600_) of the pre-cultured broth was determined by using a Biotek Synergy HT spectrophotometer, and then the pre-cultured broth was inoculated into tubes containing 3 mL of yeast minimal medium (YMM) and brought to an OD_600_ reading of 0.02. YMM consisted of 6.7 g L^-1^ yeast nitrogen base without amino acids (Difco, Beijing), 20 g L^-1^ dextrose and one type of stressor. The stressors applied were either 10% (v/v) of ethanol, 42°C (heat) or 2 M of KCl (osmotic stress), respectively. Strains grown in YMM broth without stressors were used as controls and incubated at 30°C with shaking at 200 rpm for 72 h. All experiments were performed in duplicate and repeated at least three times.

### Biomass dry weight determination

To determine the biomass dry weight (in mg) of stress cultures, 3 mL of sample suspensions was transferred into weighed dry Eppendorf tubes and centrifuged for 5 min at 13400 g. The pellets were washed twice with distilled water. Then, the pellets were dried for 8 h at 105°C after which they were weighed by using a Mettler Toledo XS105 Dual Range Analytical Balance (Schweiz, Readability = 0.01 mg).

### Stress tolerance analysis

To investigate stress tolerance variations of strains, we initially evaluated three levels for each of the stressors (ethanol: 8% (v/v), 10%, or 12%; heat: 40°C, 42°C, or 45°C; osmotic stress: 1 M, 2 M, or 3 M of KCl). The highest divergence was represented at middle stress levels for all stressors and thus the comparison of stress tolerance was performed by using these conditions (10% ethanol, 42°C, and 2 M of KCl). The stress tolerance of strains was evaluated by biomass dry weight after growth in stressful media. The comparison analyses among different groups were performed by using the Analysis of Variance (ANOVA) method and Nonparametric Tests. For independent samples (k, k>2), One-way ANOVA including Homogeneity of Variance Test and Least-Significant Difference (LSD) was valid when equal variance was assumed. Kruskal-Wallis test as an efficient Nonparametric Tests method was used when equal variance was not assumed. The correlations between the variability of stress tolerance and ecological sources or geographic locations were estimated by the Pearson correlation coefficient r. Strains were categorized into sub-groups according to ecological sources or geographic locations. The stress tolerance (biomass) of strains between sub-groups was compared and the Pearson correlation coefficient was calculated by ANOVA analysis. The hierarchical clustering of phenotypes was performed using Cluster 3.0 software [29].

## Results and Discussion

### Population genetic variation

Genetic variation was surveyed in 121 strains of *S*. *cerevisiae* using a dataset including 6519 bp of DNA sequence. The *S*. *cerevisiae* populations consist of 71 Chinese natural isolates, 12 industrial strains from CGMCC, and 38 SGRP isolates (Table 1) [11,12]. A total of 227 SNPs were identified, constituting 93 haplotypes. Heterozygous sites were found in 8 of 83 strains (SGRP isolates excluded) and at 45 SNPs. The natural isolates displayed a lower heterozygote to homozygote ratio of 5.6% (4/71) compared with the industrial strains representing that of 33.3% (4/12). Most natural isolates are homothallic diploids and haploid spores are capable of switching mating type and selfing, which may facilitate the remove of heterozygosity [14].

Neutral evolution was estimated in the 7 gene loci as described in the Material and Method section by the McDonald & Kreitman (MK) test [25]. Fixations were inferred from comparison of *S*. *cerevisiae* and the closest outgroup, *S*. *paradoxus*. The MK test did not reject neutral evolution for *ACT1*, *HSP104*, *RPB1*, *RPN2*, and TBP, while rejected neutral evolution for *HXK1* and *TPS1* due to an excess of replacement polymorphism than expected under neutral evolution (Table 2). To determine whether synonymous sites in *HXK1* and *TPS1* were affected by selection, codon bias was calculated and the number of unpreferred and preferred changes was compared along the lineage leading to *S*. *cerevisiae* and within strains of *S*. *cerevisiae*. In *S*. *cerevisiae*, those genes with expected neutral synonymous sites had a high effective number of codons (ENC > 45) and an equal number of preferred to unpreferred (P to U) and unpreferred to preferred (U to P) changes [30]. An ENC of 49.8 and equal P to U and U to P were exhibited in *TPS1* for both polymorphic and fixed synonymous changes, in line with the expectation for neutral sites. *HXK1* showed an ENC of 35.3 and a significant nonequilibrium on polymorphic changes (18:3 for P to U versus U to P), indicating that the synonymous sites in *HXK1* were not effectively neutral. The *HXK1* gene encodes the hexokinase isoenzyme I. *HXK1* gene was previously reported locating in quantitative trait loci related to ethanol resistance and it was upregulated after ethanol shock [20,21]. *TPS1* is a necessary gene for trehalose biosynthesis in *S*. *cerevisiae*, and trehalose is a major reserve carbohydrate involved in responses to thermal, osmotic, oxidative, and ethanol stresses [18]. The function of *HXK1 and TPS1* in stress response may correlate with the evolution of them.

**Table 2:** McDonald–Kreitman Tests of Neutrality.

Gene	P-value	Number of alleles		Polymorphic		Fixed	
		S.cer	S. par	syn	nonsyn	syn	nonsyn
ACT1	\	121	36	19	0	9	0
HSP104	0.154	121	36	59	5	40	0
HXK1	0.00777	121	36	99	25	58	3
RPB1	0.127	121	22	26	5	40	2
RPN2	0.14	121	36	50	9	47	3
TBP	0.0768	121	36	41	6	29	0
TPS1	0.00227	121	36	72	13	88	2

### Population structure

**Fig 1:**
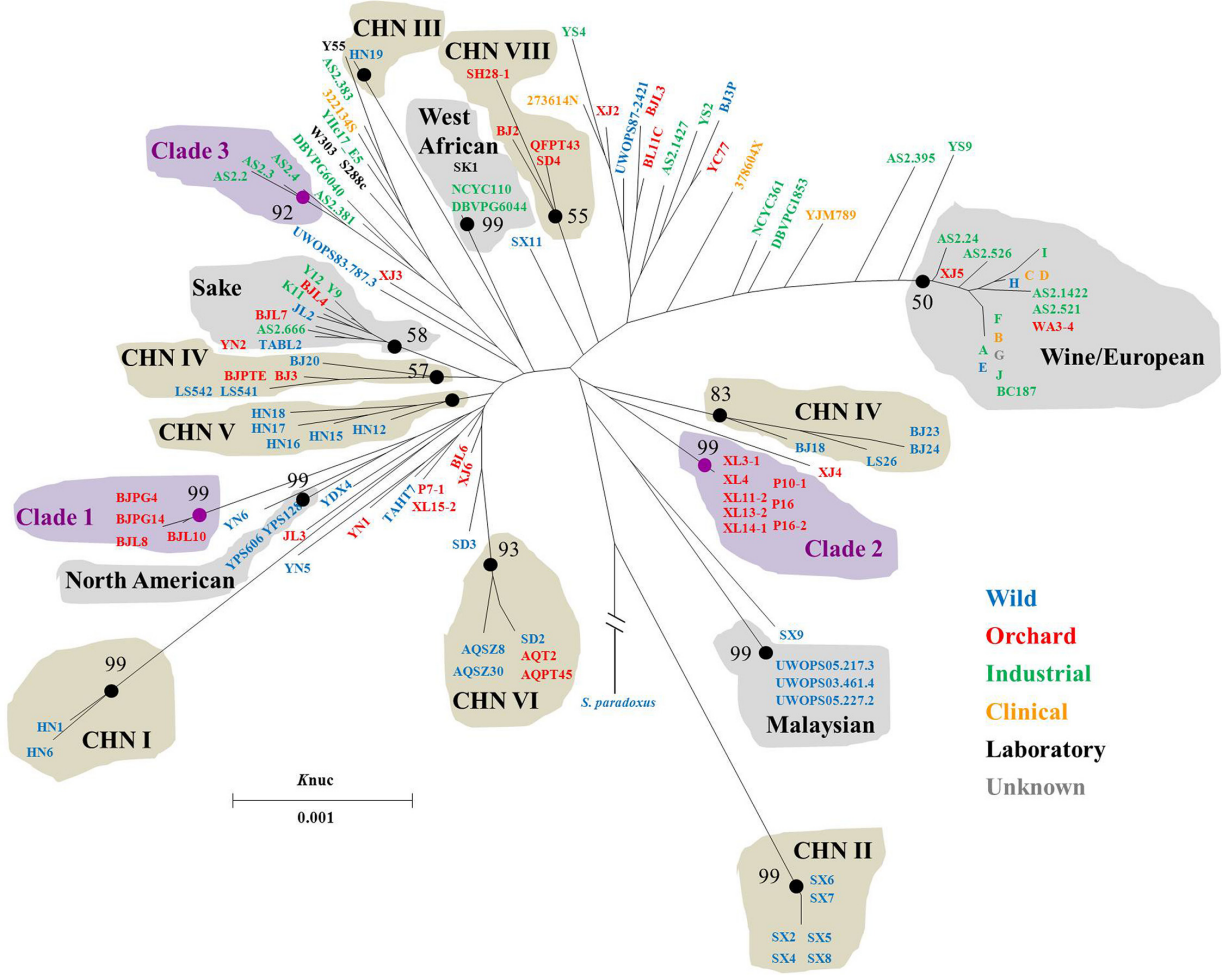
Neighbor-joining tree constructed from concatenated sequences of gene loci, for which neutral evolution was not rejected. Bootstrap percentages over 50% from 1000 bootstrap replicates were shown. *Saccharomyces paradoxus* was used as the out-group. Light grey shadow denotes the world lineages, tawny shadow denotes the Chinese lineages, and purple shadow denotes the Clades proposed in this study.

Phylogenetic relationships among strains of *S*. *cerevisiae* were inferred from a neighbor-joining (NJ) tree constructed from concatenated sequences of gene loci, for which neutral evolution was not rejected, namely *ACT1*, *HSP104*, *RPB1*, *RPN2*, TBP, and the synonymous sites in *TPS1*. For the strains containing heterozygous sites, one randomly selected haplotype was used in the analysis. Seven of the eight Chinese lineages and the five world lineages previously proposed were depicted in the NJ tree (Fig 1). The strains in Chinese lineage CHN IV were separated into two clades in this study, which should be due to the used gene loci different from previous study for phylogenetic analysis [11]. In addition to the previous reported lineages, three other clades with strong bootstrap values (>92%) were identified and designated as Clade 1, Clade2, and Clade 3 (Fig 1). The Clade 1 and Clade2 strains were isolated from fruits in northern China and western China, respectively, while the Clade3 strains constituted beer yeasts from England (Table 1). Structure analysis supported the general groupings of the strains (S1 Fig). Additional gene loci or genome sequences are needed to estimate if the three Clades are novel *S*. *cerevisiae* lineages.

### Population stress tolerance

The strains used for stress tolerance analysis include 71 Chinese natural isolates and 12 industrial strains (Table 1). Growth lag, growth rate and growth efficiency are the key variables of cellular growth dynamics [31]. Growth dynamics of representative strains showed a longer growth lag, a smaller growth rate, and lower growth efficiency under stress conditions compared with the controls without stress during the tests for 116 hours, indicating the feasibility of these variables used to reflect stress tolerance (S2 Fig). The growth efficiency (biomass) for 72 h was used to infer stress tolerance in this study. Because ethanol and heat stress lead to cell aggregation and flocculation, which might cause a biased and unreliable measurement of cell density, biomass dry weight of cultures instead of optical density was determined. Bivariate correlation analysis between the data in duplicate represented a high correlation at p<0.001 for each stressors (Heat, Pearson r = 0.726; ethanol, Pearson r = 0.83; osmotic stress, Pearson r = 0.905) supporting the reliability of the measurement.

**Fig 2:**
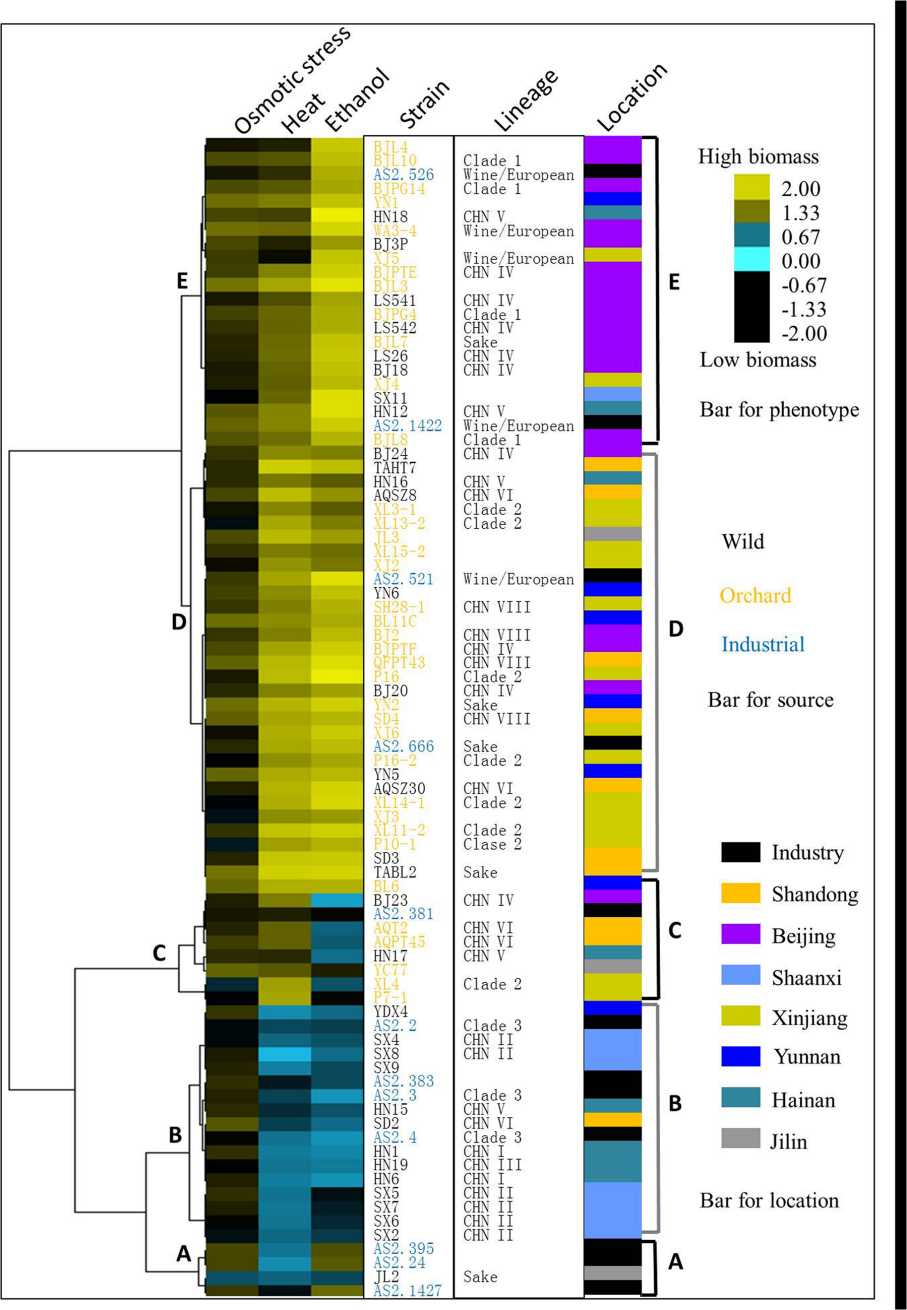
Stress tolerance variations in the *S*. *cerevisiae* isolates. Hierarchical clustering of phenotypes was performed using a centered Pearson correlation metric and average linkage mapping. Biomass of strains cultured for 72h in stressful conditions was used to infer stress tolerance. Locations of the isolates were also depicted. The industrial strains from multiple locations were not subdivided.

Hierarchical clustering of the 83 strains of *S*. *cerevisiae* was performed according to biomass dry weight data (Fig 2). Five clusters were recognized and designated as A to E. Most strains (40/60) in an identical genetic lineage were grouped in an identical phenotypic cluster. All isolates in lineages CHN I, CHN II, CHN III, and Clade 3 gathered in cluster B; all isolates in lineage CHN VIII and Clade 1 gathered in cluster D and E, respectively; The Clade 2 isolates excluding XL4 were placed in cluster D. Varied stress tolerance was shown in lineages CHN IV, CHN V and CHN VI, in which the isolates were placed in clusters B to E (Fig 2). These findings indicated correspondence between genotype and phenotype in lineages of *S*. *cerevisiae*.

**Fig 3:**
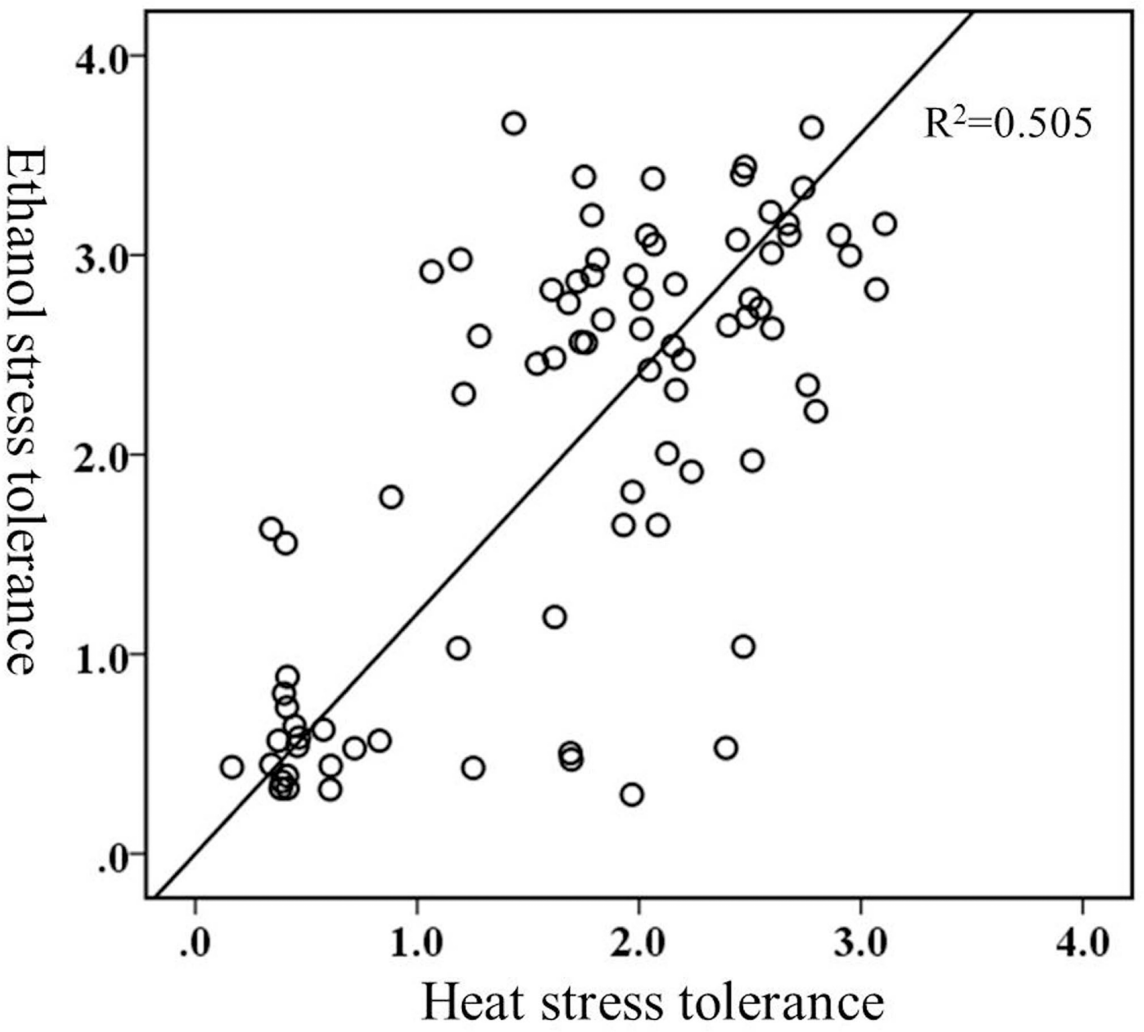
Pearson correlation between ethanol and heat stress tolerance determined by bivariate correlation analysis. Ethanol and heat stress tolerance was inferred from the biomass (gram) of 3 ml cultures grown for 72h under ethanol or heat stress.

The range of biomass variations under stresses of ethanol, heat, and osmotic stress were 0.3 to 3.7 mg, 0.17 to 3.1 mg, and 0.54 to 1.9 mg, respectively. These data displayed a larger variation on tolerance to ethanol or heat compared with osmotic stress in *S*. *cerevisiae* populations (Fig 2). Bivariate correlation analysis represented a high correlation (Pearson r = 0.71 at p<0.0001) between ethanol and heat stress tolerance (Fig 3). This finding was similar (Pearson r = 0.51 at p<0.0006) to that deduced from testing a panel of strains from vineyard, sake and clinical environments [15]. Overlapping stress responses could be induced by heat and ethanol shock, such as causing similar changes to plasma membrane protein composition and inducing heat shock proteins that contribute to both thermal and ethanol tolerance [32]. These phenomena can be explained by environmental modification of *S*. *cerevisiae* via fermentation and the subsequent selection pressures imposed on them by the production of both ethanol and heat together [33,34].

### Influences of environment on stress tolerance variations

**Fig 4:**
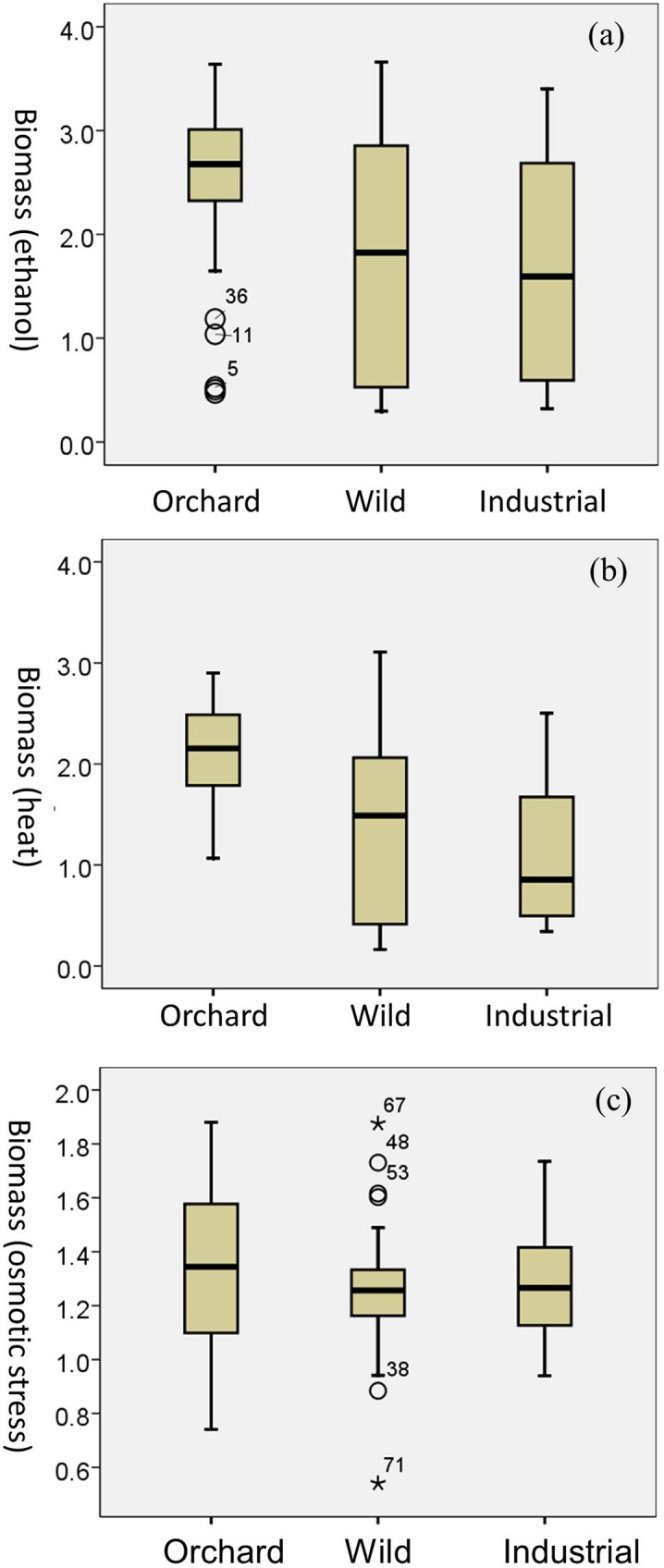
Boxplots depicting stress tolerance variations between the ecological groups. (a) ethanol stress; (b) heat stress; (c) osmotic stress. The number of strains in the Orchard, Wild, and Industrial group was 37, 34, and 12, respectively. The box contains the middle 50% of the data. Horizontal line inside the box: median; Upper boundary of whisker: largest observed value that is not an outlier; Lower boundary of whisker: smallest observed value that is not outlier. ○ = values >1.5 box-lengths from the box but not extremes (outliers). * Values >3 box-lengths from the box (extremes).

To evaluate the impact of ecological source on stress tolerance variations of *S*. *cerevisiae* strains, the 83 strains were categorized into three groups based on ecological sources, namely Orchard, Wild, and Industrial (Table 1). Stress tolerance was compared between the three groups. Significantly higher levels of tolerance to ethanol (ANOVA, at the 0.05 level) and heat stress (Kruskal-Wallis Test, at the 0.05 level) presented in the Orchard group compared with the Wild and Industrial groups, indicating the influence of ecological source on phenotypic variations of *S*. *cerevisiae* strains (Fig 4A and 4B). Tolerance to osmotic stress was comparable among the three groups (Fig 4C). Unexpectedly, the Industrial group did not overall generate a high stress tolerance level. This is because the Clade 3 and mosaic strains in the Industrial group represented stress sensitivity, though the Sake and Wine/European strains from distilled spirit in the Industrial group showed tolerance to ethanol and heat stress (Table 1 and Fig 2).

**Fig 5:**
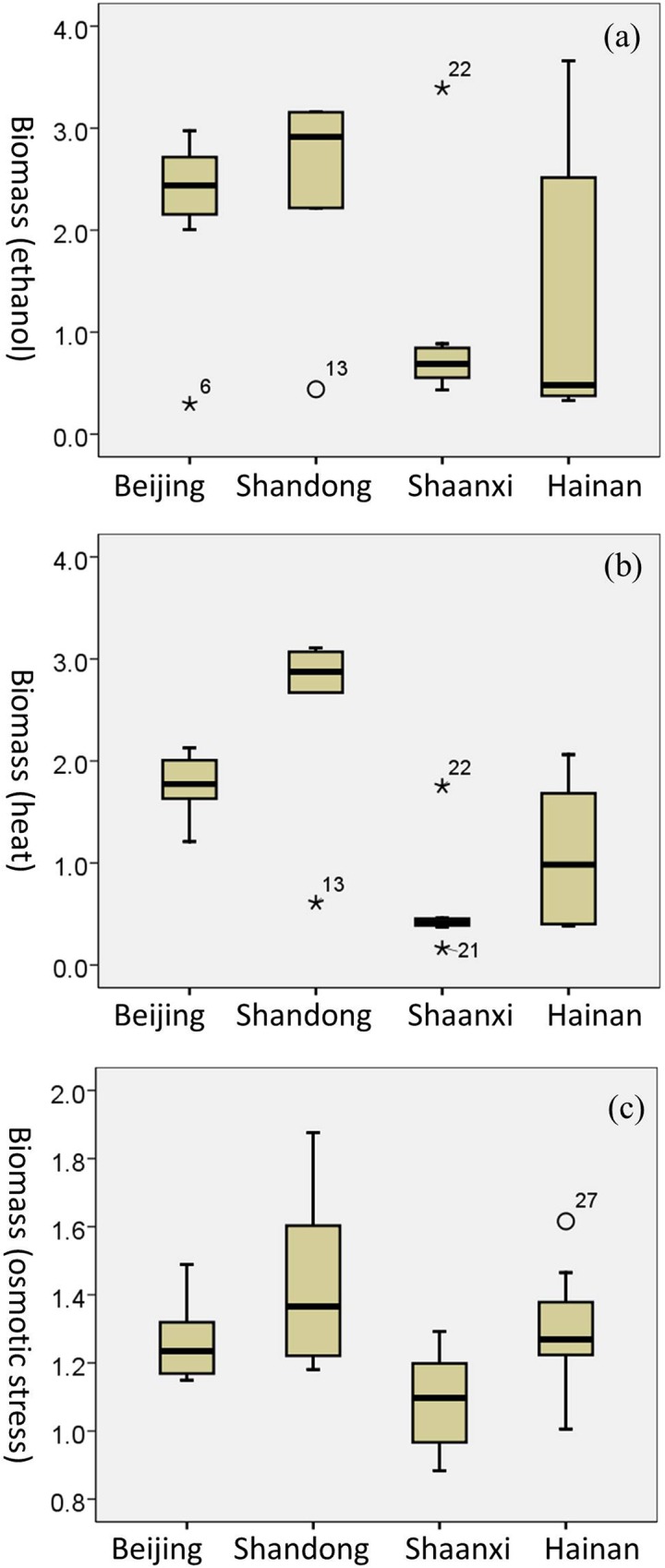
Boxplots depicting stress tolerance variations in the Wild group. (a) ethanol stress; (b) heat stress; (c) osmotic stress. ○ = values >1.5 box-lengths from the box but not extremes (outliers). * Values >3 box-lengths from the box (extremes).

**Fig 6:**
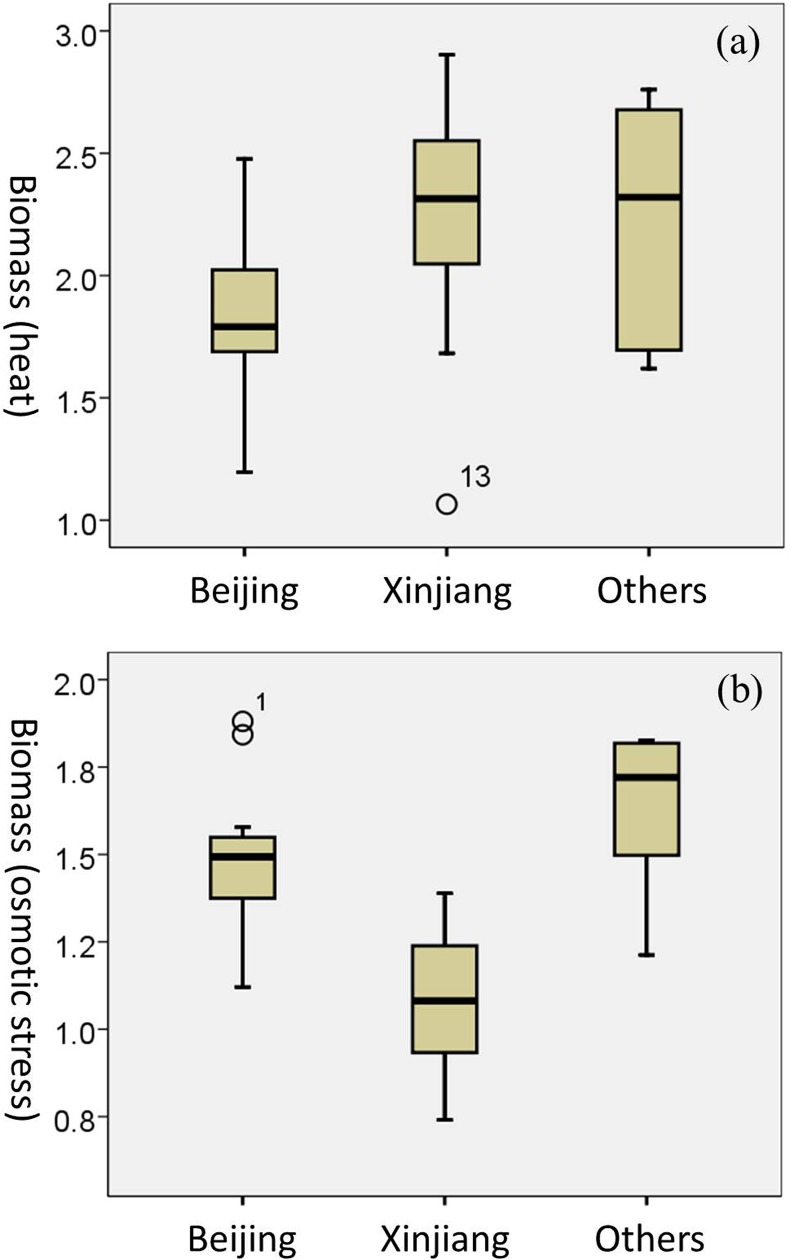
Boxplots depicting stress tolerance variations in the Orchard group. (a) heat stress and (b) osmotic stress. Ethanol stress data was not shown because no significant difference was presented among sub-groups. ○ = values >1.5 box-lengths from the box but not extremes (outliers). * Values >3 box-lengths from the box (extremes).

To investigate influences of geographic location on stress tolerance variations, strains in an identical ecological group were divided into sub-groups based on geographic locations (provinces) (Table 1 and S3 Fig). Significant differences (ANOVA, at the 0.05 level) presented among the sub-groups in both the Wild group and the Orchard group (Fig 5 and Fig 6). For example, the Wild strains isolated from Beijing and Shandong province displayed a significantly (ANOVA, at the 0.01 level) higher level of tolerance to ethanol and heat stress compared with those Wild strains (lineage CHN II) from Shaanxi province (Fig 5). The Pearson correlation coefficient (r) to the variability of ethanol and heat stress was 0.51 and 0.77, from which 25.67% and 59.98% of the variability could be explained by geographic locations, respectively. The Orchard isolates from Xinjiang province presented significantly (ANOVA, at the 0.05 level) higher sensitivity to osmotic stress compared with those Orchard isolates in other sub-groups (Fig 6). The Pearson correlation coefficient to the variability of osmotic stress was 0.78, from which 60.73% of the variability could be explained by geographic locations. These results indicated the correlation between phenotypic variations of *S*. *cerevisiae* strains and geographic location.

Overall, both ecological source and geographic location showed close correlation to the phenotypic variations of *S*. *cerevisiae* populations. In the evolutionary history, *S*. *cerevisiae* has interacted with insects and/or human activities, which may help to shape the ecology and geography of the yeast [35,37]. In this study, most of the isolates from human-associated environments possess higher levels of stress tolerance compared with those from settings spared anthropogenic influences. The lineages from Shandong province and Beijing areas (CHN VIII and Clade 1) showed high levels of stress tolerance, while the lineage from Shaanxi province (CHN II) and most isolates from Hainan province (CHN V) were sensitive to stressors (Fig 2 and S3 Fig). Beijing, Shandong and Shaanxi provinces are all located in northern China and have a temperate climate (S3 Fig). Hainan province is a tropical island in southern China. This information indicated that climatic zones are not closely related to stress tolerance variations in *S*. *cerevisiae*. The strains from Shaanxi (CHN II) and Hainan province (CHN V) were all isolated in primeval forests spared anthropogenic influences, like its closest wild relative, *S*. *paradoxus* [11,37,38]. The strains from Shandong province and Beijing areas (CHN VIII and Clade 1) were isolated in orchards, which are human associated environments. It seems that human activities have exerted strong influences on stress tolerance variations in *S*. *cerevisiae*. This viewpoint achieved supports from this study and previous publications [11,36,37,39–41]. The evidence from study of Chinese lineages was described as follows. First, *S*. *cerevisiae* population migration was previously observed between different Chinese geographic locations and from primeval forests to human-associated environments [11]. Second, the Orchard group represented significantly higher level of tolerance to ethanol and heat stress than the Wild group in this study (Fig 4). Third, the Chinese lineage CHN VIII displaying a high level of stress tolerance consisted of strains isolated from orchards in three different Chinese geographic regions, while the stress sensitive Chinese lineages CHN I, II, or III consisted of strains from primeval forests (Table 1, Figs 1 and 2). Fourth, the domestic Sake and Wine/European lineages also contained Chinese isolates and most of them represented higher levels of stress tolerance than those from primeval forests (Figs 1 and 2).

Industrial bioethanol yeast strains are generally considered possessing strong tolerance to stressors. Nevertheless, there are natural *S*. *cerevisiae* strains can outcompete industrial bioethanol strains in tolerance to specific stressors [42,43]. In our previous study, we found that the strain JZ1C in Orchard group showed high tolerance to heat and this strain could efficiently converse inulin into ethanol at 40°C [44]. These findings indicated the possibility to isolate novel superior *S*. *cerevisiae* strains for ethanol fermentation from nature.

## Conclusions

In this study, we compared the stress tolerance of diverse *S*. *cerevisiae* strains and estimated the influence of ecology and geography on variations of stress tolerance in the yeast. Both ecological sources and geographical locations presented significant correlations with variations of stress tolerance, which might be explained by the influence of human activities on the evolutionary history of *S*. *cerevisiae*. Overall, the isolates from human-associated environments represented a higher level of stress tolerance compared with those from forests spared anthropogenic influences. This study provides guidelines for selection of robust *S*. *cerevisiae* strains for bioethanol production from nature.

## Supporting Information

S1 Fig Population structure of *S*. *cerevisiae* inferred from 143 SNPs using the program Structure (version 2.3.2) under different K values (the numbers of populations assumed). Admixture model with correlated allele frequencies was used. Both the length of burnin and the MCMC repetitions after burnin were 100,000. Each strain is represented by a single vertical line broken into K colored segments, with lengths proportional to each of the K inferred clusters.
(DOCX)

S2 Fig Growth curve of representative strains under stress and control conditions. YMM cultures of 3 ml were weighed at each selected time point. Stressors: ethanol, 10% (v/v); heat, 42°C; osmotic stress, 2 M of KCl. All of the experiments were performed in triplicate. Standard deviations (error bars) are shown.
(DOCX)

S3 Fig The geographic locations of the strains isolated in China. A, Xinjiang province in Northwest China; B, Shaanxi province in the Loess Plateau; C, Yunnan province in Southwest China; D, Hainan province in Maritime South China; E, Shandong province in North China Plain; F, The suburb of Beijing in North China Plain; G, Jilin province in Northeast China.
(DOCX)

S1 Table Gene loci used in this study and primers for PCR. (DOCX)
