# Supplementary material for: Stress Tolerance Variations in Saccharomyces cerevisiae Strains from Diverse Ecological Sources and Geographical Locations
Source: PLoS One. 2015 Aug 5;10(8):e0133889. doi: 10.1371/journal.pone.0133889 (PMC4526645; doi:10.1371/journal.pone.0133889)
Supplement: S1 Fig — Admixture model with correlated allele frequencies was used. Both the length of burnin and the MCMC repetitions after burnin were 100,000. Each strain is represented by a single vertical line broken into K colored segments, with lengths proportional to each of the K inferred clusters. (DOCX) [file pone.0133889.s001.docx]

**
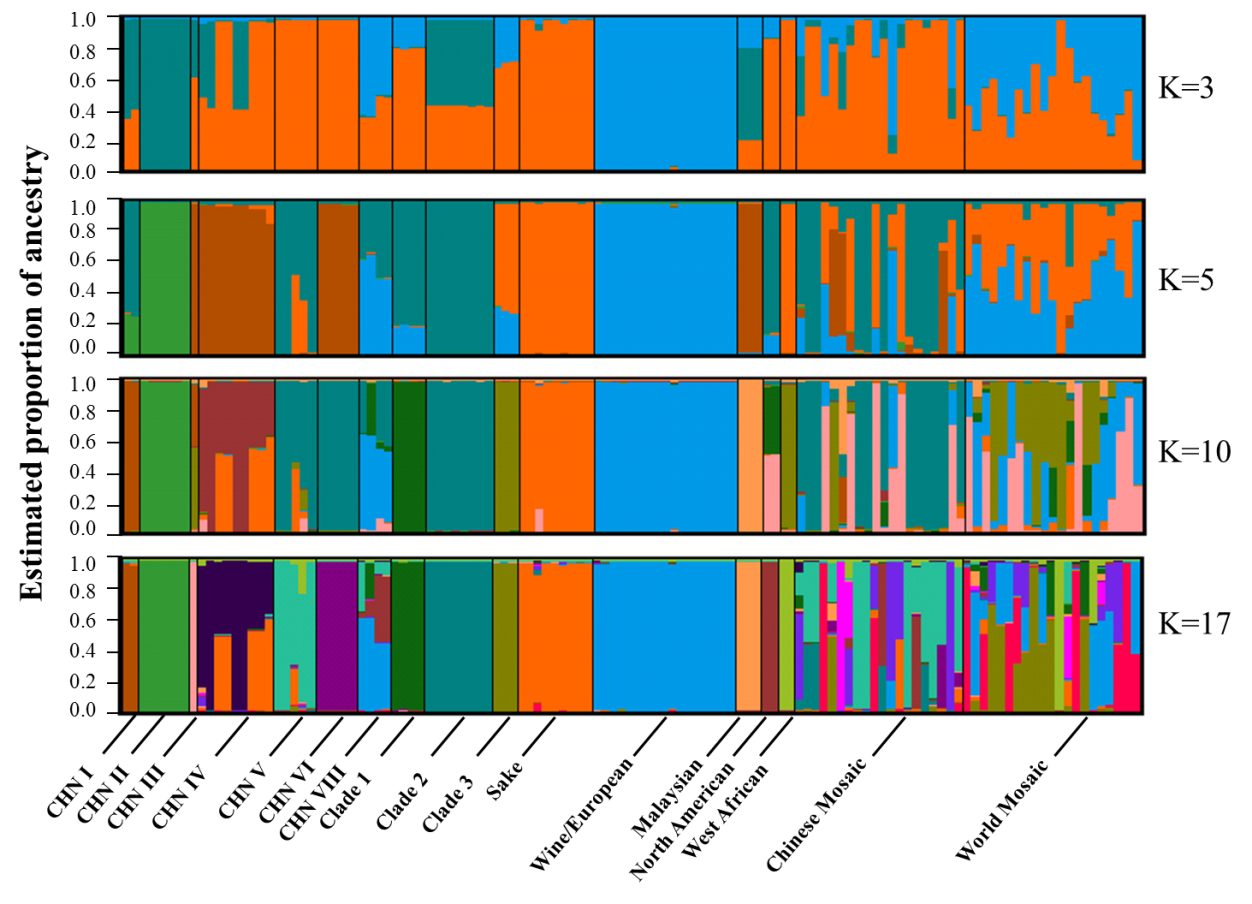
**

**S1 Fig.** Population structure of *S. cerevisiae* inferred from 143 SNPs using the program Structure (version 2.3.2) under different K values (the numbers of populations assumed). Admixture model with correlated allele frequencies was used. Both the length of burnin and the MCMC repetitions after burnin were 100,000. Each strain is represented by a single vertical line broken into K colored segments, with lengths proportional to each of the K inferred clusters.
