# Supplementary material for: Stress Tolerance Variations in Saccharomyces cerevisiae Strains from Diverse Ecological Sources and Geographical Locations
Source: PLoS One. 2015 Aug 5;10(8):e0133889. doi: 10.1371/journal.pone.0133889 (PMC4526645; doi:10.1371/journal.pone.0133889)
Supplement: S2 Fig — YMM cultures of 3 ml were weighed at each selected time point. Stressors: ethanol, 10% (v/v); heat, 42°C; osmotic stress, 2 M of KCl. All of the experiments were performed in triplicate. Standard deviations (error bars) are shown. (DOCX) [file pone.0133889.s002.docx]

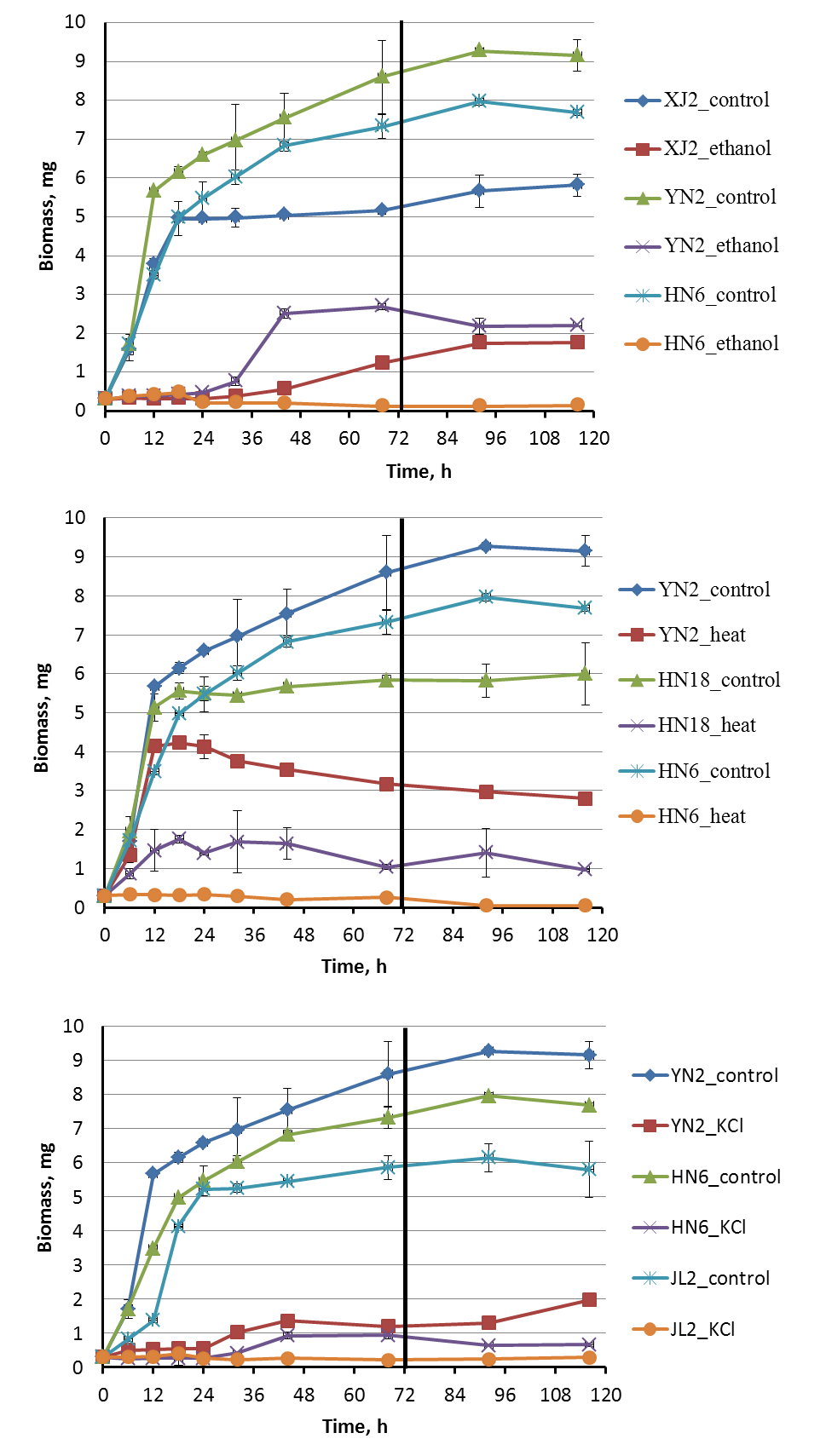


**S2** **Fig.** Growth curve of representative strains under stress and control conditions. YMM cultures of 3 ml were weighed at each selected time point. Stressors: ethanol, 10% (v/v); heat, 42 ºC; osmotic stress, 2 M of KCl. All of the experiments were performed in triplicate. Standard deviations (error bars) are shown.
