# Supplementary material for: Stress Tolerance Variations in Saccharomyces cerevisiae Strains from Diverse Ecological Sources and Geographical Locations
Source: PLoS One. 2015 Aug 5;10(8):e0133889. doi: 10.1371/journal.pone.0133889 (PMC4526645; doi:10.1371/journal.pone.0133889)
Supplement: S3 Fig — A, Xinjiang province in Northwest China; B, Shaanxi province in the Loess Plateau; C, Yunnan province in Southwest China; D, Hainan province in Maritime South China; E, Shandong province in North China Plain; F, The suburb of Beijing in North China Plain; G, Jilin province in Northeast China. (DOCX) [file pone.0133889.s003.docx]

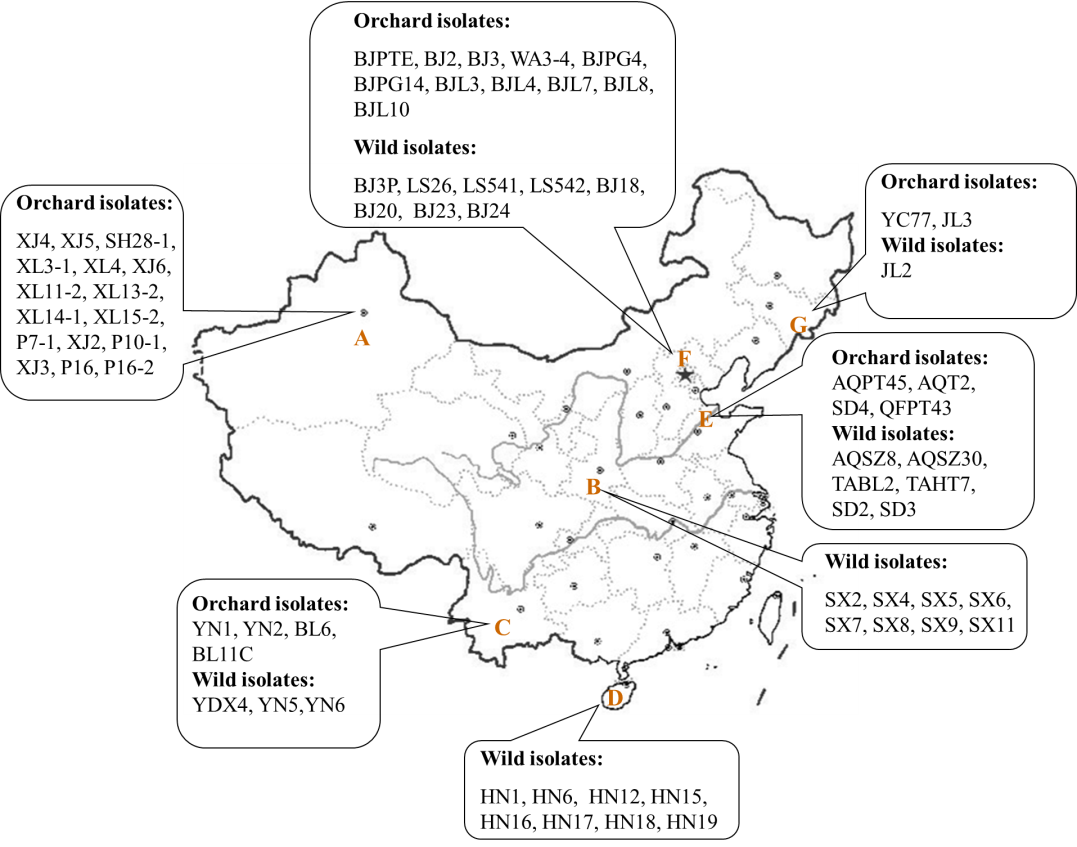


**S3 Fig.** The geographic locations of the strains isolated in China. A, Xinjiang province in Northwest China; B, Shaanxi province in the Loess Plateau; C, Yunnan province in Southwest China; D, Hainan province in Maritime South China; E, Shandong province in North China Plain; F, The suburb of Beijing in North China Plain; G, Jilin province in Northeast China.
