# Supplementary material for: Stress Tolerance Variations in Saccharomyces cerevisiae Strains from Diverse Ecological Sources and Geographical Locations
Source: PLoS One. 2015 Aug 5;10(8):e0133889. doi: 10.1371/journal.pone.0133889 (PMC4526645; doi:10.1371/journal.pone.0133889)
Supplement: S1 Table — (DOCX) [file pone.0133889.s004.docx]

**S1 Table.** Gene loci used in this study and primers for PCR.

| Gene locus | Chromosome | Primer sequence | Size of amplicon, bp |
| --- | --- | --- | --- |
| ACT1 | VI | TACCCAATTGAACACGGTAT | 564 |
|  |  | TCTGAATCTTTCGTTACCAAT |  |
| *HSP104* | XII | GCTTCGGATCATCAACAT | 826 |
|  |  | CGTCGTCCTTACCATTAC |  |
| *HXK1* | II | AGAGCAATAAGAAACAATTGTGG | 1639 |
|  |  | AAAACATA AGGGCATCACTCAT |  |
| RPB1 | IV | TCAAACTTGTCAAGAGGGTATG | 903 |
|  |  | TTTGGAACACCGACTTGGTCTA |  |
| RPN2 | IX | AGCATGGCAATATTACTCGT | 875 |
|  |  | TGCTACCTTCTCTACCTCCTT |  |
| *TBP* | V | TACTAGTTAGACTGCTCTGC | 841 |
|  |  | TACTCCTTCCCCA TCACA |  |
| *TPS1* | II | GTACAGCCGGGTGGTAGAG | 1762 |
|  |  | GTGTGGCGTTTCAGTTATG |  |
